# Platelet-Activating Factor Receptor Plays a Role in Lung Injury and Death Caused by Influenza A in Mice

**DOI:** 10.1371/journal.ppat.1001171

**Published:** 2010-11-04

**Authors:** Cristiana C. Garcia, Remo C. Russo, Rodrigo Guabiraba, Caio T. Fagundes, Rafael B. Polidoro, Luciana P. Tavares, Ana Paula C. Salgado, Geovanni D. Cassali, Lirlândia P. Sousa, Alexandre V. Machado, Mauro M. Teixeira

**Affiliations:** 1 Departamento de Bioquímica e Imunologia, Instituto de Ciências Biológicas, Universidade Federal de Minas Gerais, Belo Horizonte, MG, Brazil; 2 Centro de Pesquisas René Rachou, Fundação Oswaldo Cruz, Belo Horizonte, MG, Brazil; 3 Departamento de Patologia, Instituto de Ciências Biológicas, Universidade Federal de Minas Gerais, Belo Horizonte, MG, Brazil; 4 Departamento de Análises Clínicas e Toxicológicas, Faculdade de Farmácia, Universidade Federal de Minas Gerais, Belo Horizonte, MG, Brazil; Erasmus Medical Center, Netherlands

## Abstract

Influenza A virus causes annual epidemics which affect millions of people worldwide. A recent Influenza pandemic brought new awareness over the health impact of the disease. It is thought that a severe inflammatory response against the virus contributes to disease severity and death. Therefore, modulating the effects of inflammatory mediators may represent a new therapy against Influenza infection. Platelet activating factor (PAF) receptor (PAFR) deficient mice were used to evaluate the role of the gene in a model of experimental infection with Influenza A/WSN/33 H1N1 or a reassortant Influenza A H3N1 subtype. The following parameters were evaluated: lethality, cell recruitment to the airways, lung pathology, viral titers and cytokine levels in lungs. The PAFR antagonist PCA4248 was also used after the onset of flu symptoms. Absence or antagonism of PAFR caused significant protection against flu-associated lethality and lung injury. Protection was correlated with decreased neutrophil recruitment, lung edema, vascular permeability and injury. There was no increase of viral load and greater recruitment of NK1.1^+^ cells. Antibody responses were similar in WT and PAFR-deficient mice and animals were protected from re-infection. Influenza infection induces the enzyme that synthesizes PAF, lyso-PAF acetyltransferase, an effect linked to activation of TLR7/8. Therefore, it is suggested that PAFR is a disease-associated gene and plays an important role in driving neutrophil influx and lung damage after infection of mice with two subtypes of Influenza A. Further studies should investigate whether targeting PAFR may be useful to reduce lung pathology associated with Influenza A virus infection in humans.

## Author Summary

Influenza virus causes disease that affects people from different age, gender or social conditions. The illness spreads easily and affects millions of people every year. Vaccines are effective preventive approaches, but the high degree of viral antigenic drift requires annual formulation. Anti-viral drugs are used as therapy, but are only effective at the very early stages of disease. The main symptoms that lead to hospitalizations and deaths are associated with the severe inflammatory host immune response triggered by the virus infection. Our approach was to decrease the inflammatory events associated with the viral infection by targeting a molecule, Platelet Activating Factor receptor (PAFR), known to induce several inflammatory events, including leukocyte recruitment and leakage. We found that PAFR deficient mice or wild type mice treated with a PAFR antagonist had less pulmonary inflammation, pulmonary injury and lethality rates when infected by two subtypes of Influenza A virus. In contrast, the immune response against the virus, as assessed by viral loads and specific antibodies, were not decreased. Our findings concur with the idea that severe inflammation plays an important role in flu morbidity and mortality and show that PAFR is a major driver of the exacerbated inflammation in mice infected with Influenza A virus.

## Introduction

Influenza A viruses belong to the *Orthomixoviridae* family of RNA single-stranded, negative-sense viruses and cause epidemics, leading to about 250,000 to 500,000 deaths and 3 to 5 million severe cases annually worldwide [1], [2]. The new Influenza A H1N1 pandemic and the warning of a possible avian H5N1 pandemic increased the search for new vaccines and therapies [2], [3]. Antiviral drugs are an attractive possibility [4]. However, the need for the initiation of treatment very early in the course of infection [5] and the possibility of resistance suggest that novel alternatives are necessary [6]. A promising approach to reduce flu morbid is targeting immune molecules and cells related to disease severity (reviewed in [7]).

The immune system is activated shortly after respiratory epithelial cells have been infected by Influenza A. The single stranded RNA of Influenza virus is sensed inside endosomes by Toll like receptor 7 (TLR7) and TLR8 [8] and in cytoplasm by the helicase RIG-I (retinoic acid inducible gene-I) [9] and the inflammasome protein NLRP3 [10], [11]. TLR3 recognizes a intermediate of double strand RNA during Influenza replication [12]. The recognition of infection leads to alveolar macrophage recruitment and release of cytokines and chemokines with antiviral and proinflammatory actions (reviewed by [13]). NK cells are recruited in the first days of infection and are important for the initiation of adaptive immune responses against Influenza virus via IFN-γ production [14]. Neutrophils are another important leukocyte population involved in Influenza control [15]. Influenza A virus is a potent stimulus for neutrophil activation in the lungs and airways [16]. In addition to the antiviral action of neutrophils, excessive lung inflammation may result in lung damage, disruption of alveolar epithelial barrier and fluid leakage that limits respiratory capacity [17], [18].

Platelet Activating Factor (PAF) is a phospholipid mediator involved in many physiological and pathological conditions. The synthesis of PAF under inflammatory conditions is mediated by an acetyl-CoA:lyso-PAF acetyltransferase, named LysoPAFAT/LPCAT2 [19]. PAF acts through a single G protein-coupled receptor (PAFR) expressed in the plasma and nuclear membranes of leukocytes, endothelial cells and platelets [20]. Several inflammatory events have been associated with the administration of PAF, including increase of vascular permeability and lung edema [21], and leukocyte recruitment and activation [20], [22]. Moreover, blockade of the PAFR has been shown to decrease edema formation and/or leukocyte recruitment in several models of inflammation [23], [24], [25]. Phosphatidilcoline oxidation may also lead to the generation of PAF-like lipids that can activate the PAFR [26] and that have been reported to trigger lung injury after Influenza infection [27], [28]. Furthermore, upregulation of PAFR mRNA is seen during Influenza A virus infection [29], which is consistent with its expression on leukocytes and increase of these cells during Influenza A virus infection. Because of the involvement of PAFR during inflammatory responses and its expression during Influenza A virus infection, we hypothesized that PAFR activation may play an important role in driving pulmonary inflammation and injury in the context of Influenza A virus infection. To test our hypothesis, PAFR deficient mice or mice treated with PAFR antagonists were infected with two subtypes of Influenza A virus: a mouse-adapted Influenza virus A/WSN/33 H1N1 or a less virulent Influenza virus, H3N1. Our studies demonstrate that absence or antagonism of PAFR protects against Influenza A related lethality and inflammatory injury.

## Results

### Influenza A/WSN/33 H1N1-associated progressive weight loss, pulmonary inflammation and death

In order to determine the lethal inoculum of Influenza A/WSN/33 H1N1 virus in C57BL6/J mice, we infected the animals with four different inocula – 10^3^, 10^4^, 10^5^ and 10^6^ PFU – and accompanied them for 21 days after infection. Weight loss occurred over time after infection with 10^5^ and 10^6^ PFU and culminated with 100% death in 9 or 7 days, respectively. There was substantial weight loss in mice infected with 10^4^ PFU until the eighth day of infection; thereafter, mice which survived (45%) recovered weight gradually. There were no weight loss and deaths associated with infection with 10^3^ PFU (data not shown).

Based on the previous findings, we chose 10^4^ as the mild inoculum and 10^6^ PFU as the lethal inoculum to assess pulmonary inflammation associated with the infection. Since weight loss after infection with 10^4^ PFU was slower, we chose to evaluate lung specimens at 3-day intervals from the first to the tenth day of infection. On the other hand, shorter 2-days intervals were chosen in the experiments with 10^6^ PFU, from day one to five of infection.

**Figure 1:**
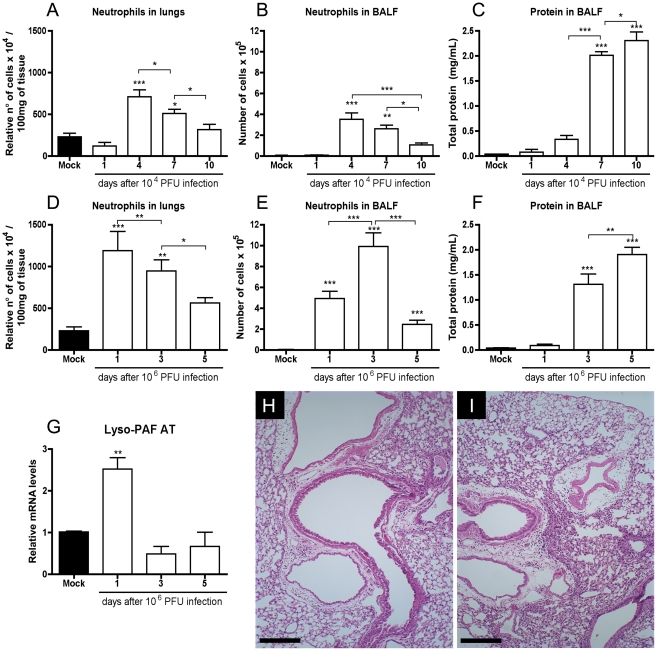
Inflammatory profile following Influenza A/WSN/33 H1N1 infection. C57BL/6J mice were infected intranasally with Influenza A/WSN/33 H1N1. Mice were killed (n = 6–8 in each group) 1, 4, 7 and 10 days after infection with 10^4^ PFU and 1, 3 and 5 days after infection with 10^6^ PFU. Relative number of neutrophils in lungs, as assessed by MPO assay, after infection with 10^4^ PFU (A) and 10^6^ PFU (D). Absolute numbers of BALF neutrophils after infection with 10^4^ PFU (B) and 10^6^ PFU (E). BALF protein leakage after infection with 10^4^ PFU (C) and 10^6^ PFU (F). Relative mRNA levels of LPAFAT/LPAFAT2 in lungs after infection with 10^6^ PF assessed by Real Time PCR (G). Representative H&E stained slides at 100× of magnification of lungs of mice at 10 days after infection (dpi) with 10^4^ PFU (H) and at 5 dpi with 10^6^ PFU mice (I); bars represent 25µm. Histology shows decreased inflammatory infiltrates and more preserved alveolar areas. Data are presented as Mean ± SEM. *, ** and *** for p<0.05, p<0.01 and p<0.001, respectively, when compared to Mock or indicated groups (one-way ANOVA, Newman-Keuls).

We found substantial accumulation of neutrophils in BALF and in lung tissue, as assessed by myeloperoxidase (MPO) activity and histology, following infection (Figure 1). After 10^4^ PFU, influx of neutrophils in the lungs (Fig 1A) and airways (Fig 1B) was first observed at day 4 after infection and returned to basal levels at day 10. When infected with the lethal inoculum (10^6^ PFU), neutrophil infiltration in the lungs and BAL fluid was already detectable at day 1 and was more pronounced than with the lower inoculum (Figure 1 D,E). Pulmonary infiltration tended to resolve by day 5 after lethal infection. Consistent with the early peak of neutrophil accumulation at one day after infection with the lethal inoculum, mRNA expression of LPAFAT/LPAFAT2, the enzyme responsible for PAF synthesis in inflammatory conditions [19], was upregulated 2.5 fold at the first day of lethal infection (Fig 1 G).

Quantification of protein in BALF may be used as a marker of plasma leakage and, consequently, of lung injury [30]. There was progressive protein accumulation in the airways after infection with both inocula (Fig 1 C, F). Lung injury could also be observed by histological evaluation in animals infected with Influenza A (Fig 1 H, I). There was peribronchiolar and perivascular infiltration at 10 days after infection with 10^4^ PFU, but, as seen by the normal thickness of alveoli, the infiltration was not present in the majority of alveolar walls (Fig 1H). Inflammation appeared to be more pronounced after infection with 10^6^ PFU with significant peribronchial, perivascular and perialveolar inflammation, with thickening of alveolar walls, edema and mucus production on bronchioles at day 5 (Fig 1I).

There was a good correlation between the levels of CXCL1 and CXCL2 in the lungs (Fig S1A, B and Fig S2A, B) and the recruitment of neutrophils (Fig 1) after infection with 10^4^ and 10^6^ PFU. This is in agreement with studies showing the role of these chemokines and their receptors for neutrophil recruitment during Influenza A virus infection [31], [32]. Levels of the chemokine CCL2 were increased above baseline from day 4 after infection with 10^4^ PFU and from day 1 after 10^6^ PFU (Fig S1C, S2C). Thereafter, levels of CCL2 remained high throughout the observation period after infection with the lethal inoculum. Levels of the pro-inflammatory cytokine TNF-α were only increased at the beginning of the infection, i.e. at days 1 and 4 after 10^4^ PFU and day 1 after 10^6^ PFU (Fig S1D, S2D).

### Decreased lethality rate and pulmonary injury in PAFR deficient mice infected with Influenza A/WSN/33 H1N1 virus

**Figure 2:**
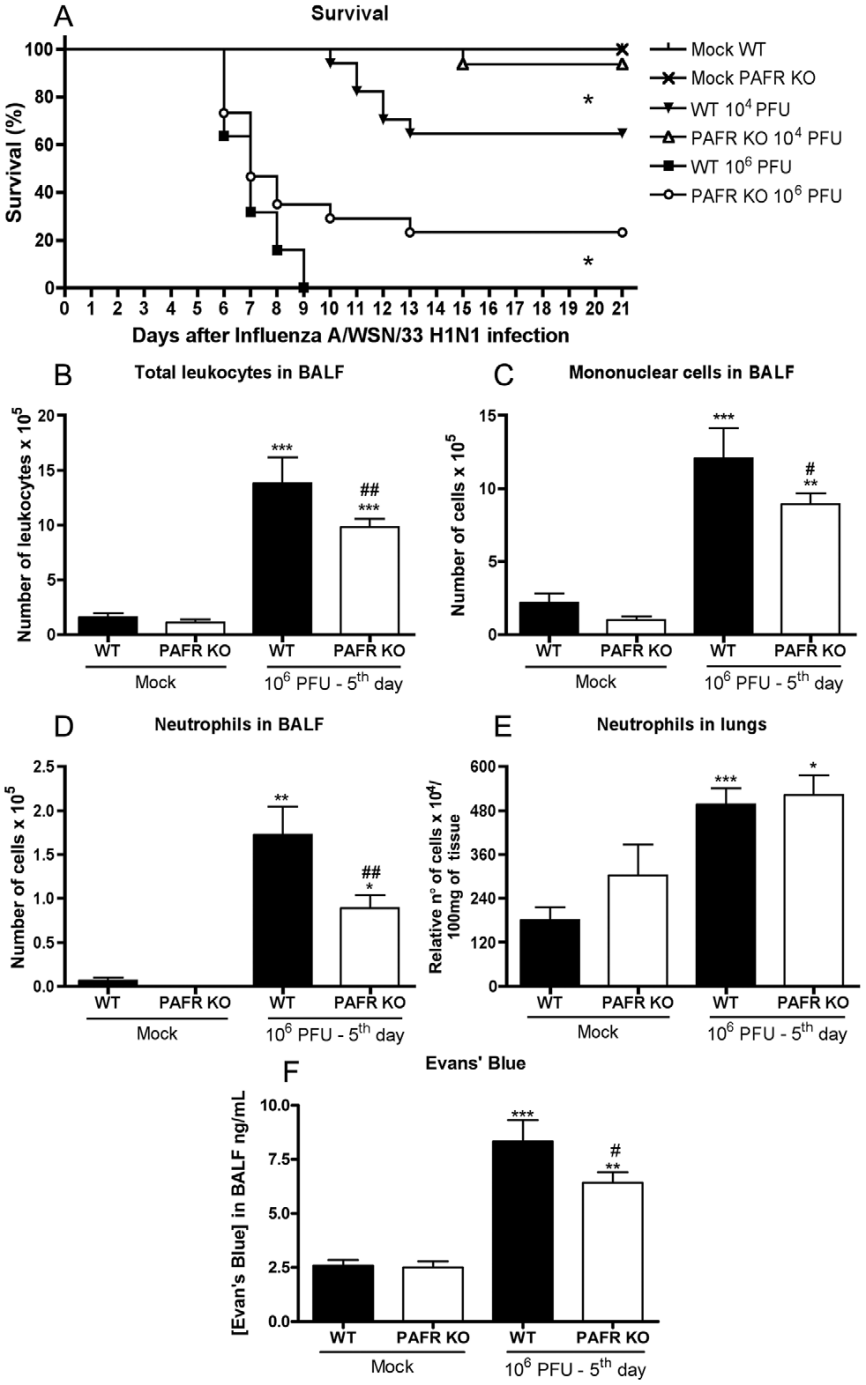
Lethal and mild Influenza A/WSN/33 H1N1 infections were less severe in PAFR KO deficient mice. WT and PAFR KO mice were challenged intranasally with 10^4^ (n = 17/16, two independent experiments) and 10^6^ PFU (n = 22/30, two independent experiments) or PBS (Mock, n = 4) and monitored for 21 days. Survival was greater in PAFR KO infected groups (A); * for p<0.05; log rank test. Total leukocyte (B), mononuclear cell (C) and neutrophil (D) influx in the airways at 5 days after infection with 10^6^ PFU Influenza A (two independent experiments). Numbers of lung neutrophils, as assessed by MPO assay (E), after infection with 10^6^ PFU of Influenza A (two independent experiments). Evans' blue concentration in BAL fluid (F) after infection with 10^6^ PFU of Influenza A was assessed at 620nm and compared to a standard curve (one experiment). Data are presented as Mean ± SEM. *, ** and *** for p<0.05, p<0.01 and p<0.001, respectively, when compared to Mock groups; # for p<0.05 and ## for p<0.01 when compared to WT infected group (one-way ANOVA, Newman-Keuls).

Aiming to evaluate a possible role of the inflammatory mediator PAF in Influenza A virus infection, PAFR and WT mice were infected with 10^4^ PFU or 10^6^ PFU of Influenza A/WSN/33 H1N1 virus. Infection of WT mice with 10^4^ PFU resulted in 35% lethality rate. In contrast, only 7% of PAFR KO mice died after infection with the same inoculum (Fig 2A). Inoculation of 10^6^ PFU caused 100% death by day 9 after infection of WT mice. In contrast, 23% PAFR KO infected with 10^6^ were alive at day 21 after infection (Fig 2A).

**Figure 3:**
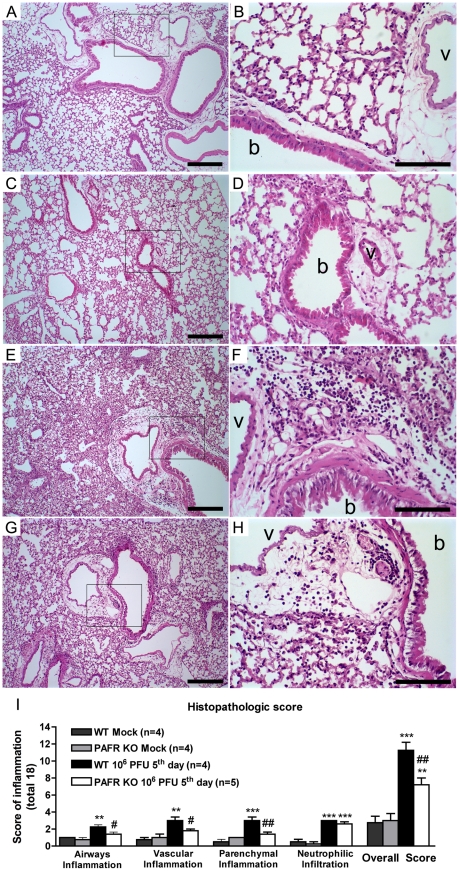
Histological changes after Influenza A WSN/33 H1N1 lethal infection in WT and PAFR deficient mice. Representative lung slides of WT mock (A, B), PAFR KO mock (C, D), WT (E, F) and PAFR deficient mice (G, H) infected with 10^6^ PFU, after five days of infection. Perialveolar infiltration, vascular and parenchyma inflammation induced by Influenza A virus infection are reduced in PAFR KO mice. Pictures on the left (A, C, E, G) were taken under 100× magnification, bars represent 25µm. Pictures on the right (B, D, F, H) were taken under 400× magnification in the areas highlighted in the lower magnification, bars represent 10µm. Bronchiole and vessels are indicated with “b” and “v”, respectively. Histopathological score (maximal of 18) evaluated airway, vascular, parenchymal inflammation, neutrophilic infiltration (I). Data are presented as Mean ± SEM. ** and *** for p<0.01 and p<0.001, respectively, compared to Mock groups; # for p<0.05 and ## for p<0.01 compared to WT infected group (one-way ANOVA, Newman-Keuls).

As the absence of PAFR resulted in partial protection from Influenza A-associated lethality and in an attempt to seek for mechanisms of protection, we evaluated several parameters of inflammation in the lungs of mice after infection with 10^6^ PFU. After infection with 10^6^ PFU, there was decreased recruitment of total leukocytes (Fig 2B), mononuclear cells (Fig 2C) and neutrophils (Fig 2D) in the airways of PAFR KO mice when compared to WT mice. Neutrophil accumulation in lungs of WT and PAFR KO infected mice was similar at the evaluated time point (Fig 2E), as assessed by MPO quantification. The concentration of Evans' blue in BALF, a marker of vascular permeability, was reduced by approximately 35% in PAFR KO when compared to WT mice infected mice (Fig 2F). To examine in greater detail pulmonary changes and inflammation induced by infection with Influenza A, lung sections stained with H&E were analyzed and graded by a pathologist blind to the experimental situations. Leukocyte infiltration into bronchioles and alveoli, hyperemia, exudation, edema and bronchial mucus production were observed in most tissue sections from infected WT mice (Fig 3E, F). Despite of similar myeloperoxidase levels (Fig 2E) and neutrophilic infiltrates (Fig 3I) in lungs of infected groups, in PAFR KO mice, cellular infiltrates were restricted to conducting airways, in peribronchiolar and perivascular areas and did not reach distal airways like alveoli and the lung parenchyma, where gas exchange occurs (Fig 3G, H). Grading scores confirmed the limitation of infiltrates around the airways, the vessels and showed that these infiltrates were present in smaller areas of the parenchyma of PAFR KO infected mice – ranging from 1 to 29% of affected parenchyma in PAFR KO infected mice against 10 to 69% of affected parenchyma in WT infected mice (p<0.05, Fig 3I). Thus, the inflammatory infiltration in the larger airways was similar in WT and PAFR KO mice infected with the lethal inoculum; however, there was more infiltration of respiratory alveoli, with thickening of alveolar walls and pneumonitis in lungs of WT mice. Experiments in animals infected with 10^4^ PFU showed decreased neutrophil influx and less protein leakage, hence less pulmonary injury, in PAFR KO mice when compared to WT infected mice after 8 days of infection (data not shown). Pulmonary inflammation after 5 days of infection with 10^4^ PFU was discrete and restricted to neutrophil infiltration and compromised a percentage of lung parenchyma between 10 and 29%, which is much less severe than lesions developed after lethal infection. Pulmonary inflammation in PAFR KO mice infected with the low inoculum was also discrete and similar to that found in lungs of WT mice (data not shown).

**Figure 4:**
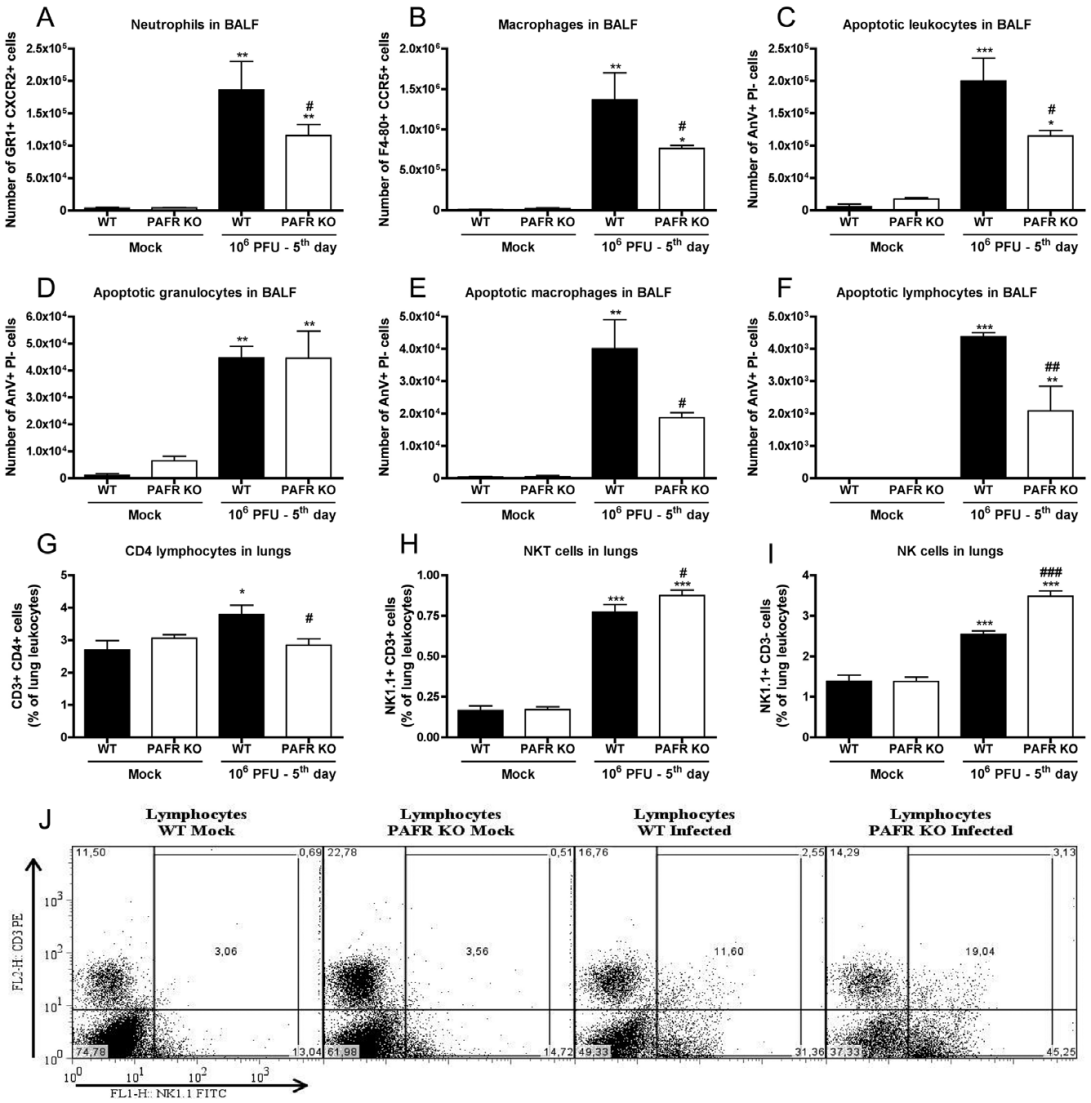
Leukocyte populations in lungs following Influenza A/WSN/33 H1N1 infection of WT and PAFR deficient mice. WT and PAFR KO mice were infected intranasally with 10^6^ PFU of Influenza A virus (n = 5/6) or PBS (n = 4) and killed after 5 days. Airway leukocytes recovered by BAL were stained with specific antibodies and evaluated by flow cytometry, according to size and granularity (A, B). Number of neutrophils GR1+ CXCR2+ (A) and macrophages F4/80+ CCR5+ (B) found in BALF. BALF cells were also stained with Annexin V (AnV) and Propidium iodide (PI) to assess apoptosis. Number of apoptotic BALF leukocytes (C), granulocytes (D), macrophages (E) and total lymphocytes (F) according to size, granularity, AnV staining and absence of PI incorporation. Leukocytes recovered from lung homogenates were also subjected to flow cytometric analysis. CD4 lymphocytes, CD3+ CD4+ (G), NKT cells, NK1.1+CD3+ (H) and NK cells, NK1.1+CD3− (I) were expressed as percentages of total leukocytes. Representative dot plots of NK1.1 and CD3 expression on lymphocytes gate (J). Data are presented as Mean ± SEM. *, ** and *** for p<0.05, p<0.01 and p<0.001, respectively, compared to Mock groups; # for p<0.05, ## for p<0.01, and ### for p<0.001, compared to WT infected group (one-way ANOVA, Newman-Keuls).

Because there was a significant change in the pathological score, we conducted a more detailed analysis of leukocyte populations in BALF and in lungs of infected mice. In the airways, there was an increase in the number of all leukocyte populations evaluated at day 5 after infection – CD4^+^ T cells, CD8^+^ T cells, NKT cells, NK cells, macrophages and neutrophils. CD8^+^ and CD4^+^ lymphocytes, NK and NKT cells were equally increased in WT and PAFR KO infected mice (data not shown). Number of granulocytes (Fig 4A) and macrophages (Fig 4B) were increased after infection of WT mice but this increase was lower in PAFR KO mice infected with the same inoculum.

We performed Annexin V binding assay to evaluate whether increased apoptosis could account for the decreased accumulation of neutrophils and macrophages in the airways after flu infection of PAFR KO mice. Influenza A induced a marked increase in the number of apoptotic cells in the airways at day 5 after infection. Overall, the number of apoptotic cells was greater in infected WT than PAFR KO mice (Fig 4C). The number of apoptotic granulocytes was similar in both infected groups (Fig 4D). Therefore, the lower number of neutrophils in the airways of PAFR KO infected mice was due to decreased recruitment of these cells and not due to a higher degree of cellular death. In contrast, there was decreased number of apoptotic macrophages and lymphocytes in infected PAFR KO than in WT mice (Fig 4E, F).

Leukocyte populations were also evaluated in lung homogenates. The percentage of lung CD3+CD8+ T cells did not increase after infection (data not shown). CD3+CD4+ T cells increased after infection in WT but not in PAFR KO mice (Fig 4G). F4-80+ CCR5+ (macrophage) and GR1+ CXCR2+ (neutrophils) cells in lung homogenates increased after infection in both WT and PAFR mice to a similar extent (data not shown). NK populations (CD3+NK1.1+ and CD3− NK1.1+) enhanced after infection in WT mice but the enhancement was significantly greater in PAFR KO mice (Fig 4H, I, J).

### Levels of cytokines and chemokines after Influenza A virus infection in WT and PAFR KO mice

**Figure 5:**
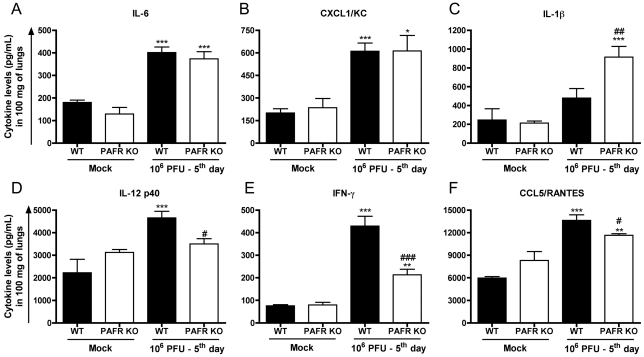
Cytokines and chemokines in lungs following Influenza A/WSN/33 H1N1 infection of WT and PAFR-deficient mice. WT and PAFR KO mice were infected intranasally with 10^6^ PFU (n = 5–9) or PBS (Mock, n = 4) and killed 5 days post infection. Pulmonary concentrations of IL-6 (A), CXCL1/KC (B), IL-1β (C), IL-12 p40 (D), IFN-γ (E) and CCL5/RANTES (F) were measured by ELISA in lung homogenates. Data are presented as Mean ± SEM. *, ** and *** for p<0.05, p<0.01 and p<0.001, respectively, compared to Mock groups; # for p<0.05, ## for p<0.01 and ### for p<0.001 when compared to WT infected group (one-way ANOVA, Newman-Keuls).

The concentrations of the cytokines IL-1β, IL-6, TNF-α, CXCL1, CXCL2, CCL5, IFN-γ and IL-12p40 were evaluated in lung homogenates of control and Influenza infected mice. As seen in Figure S2, levels of TNF-α and CXCL2 were not increased in the lungs at day 5 after infection with 10^6^ PFU infection in both WT and PAFR KO groups (data not shown). Levels of IL-6 (Fig 5A) and CXCL1 (Fig 5B) were increased after infection but there was no difference between WT and PAFR KO mice. Levels of IL-1β were also enhanced by infection but the increase was more pronounced in PAFR KO than WT infected mice (Fig 5C). Levels of IL-12p40 (Fig 5D), IFN-γ (Fig 5E) and CCL5 (Fig 5F) increased after infection and the increase was more pronounced on WT that PAFR KO mice.

### PAFR deficiency did not impair the ability of the immune system to deal with Influenza A/WSN/33 virus

**Figure 6:**
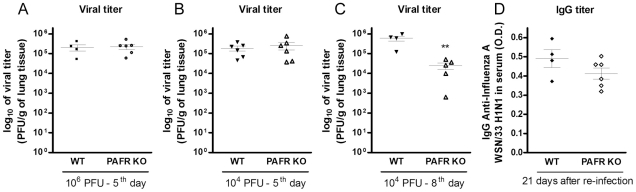
Viral load and specific antibodies after Influenza A/WSN/33 H1N1 infections of WT and PAFR-deficient mice. WT and PAFR KO mice were infected intranasally with 10^6^ PFU (n = 4–6) and killed 5 days post infection (A) or infected with 10^4^ PFU (n = 4–6) and killed 5 (B) and 8 days post infection (C). Viral titers in lungs homogenates, as assessed by MDCK plaque formation. WT and PAFR KO mice (n = 4–6) were infected intranasally with 10^3^ PFU and after 14 days were reinfected with 10^6^ PFU. After 21 days mice were killed and anti-Influenza A/WSN/33 H1N1 IgG titer was measured in serum samples by ELISA (D). Data are presented as Mean ± SEM. ** for p<0.01 when compared to WT infected group (unpaired t test).

Because pulmonary inflammation and levels of some cytokines known to be important for host resistance to viral infection, including IFN-γ and IL-12p40, were decreased in PAFR KO mice, we investigated whether this reduction was sufficient to impair viral clearance and adaptive responses. As seen in Figure 6A, there was no difference in viral load at day 5 in the lungs of WT and PAFR KO mice infected with 10^6^ PFU (Fig 6A) or 10^4^ PFU (Fig 6B). In animals infected with 10^4^ PFU, viral load was significantly lower in PAFR KO than WT mice at day 8 after infection (Fig 6C).

Next we investigated humoral adaptive immune responses in the presence or the absence of PAFR. In a re-infection protocol, animals were initially infected with a non-lethal (10^3^ PFU) inoculum of Influenza A/WSN/33 H1N1. After 14 days, animals were subjected to another infection with the same virus, at this time using the lethal inoculum, 10^6^ PFU. No weight loss or lethality was observed in WT or PAFR KO mice using this protocol (data not shown). Measurement of anti-Influenza A WSN/33 IgG revealed no differences in antibody titer between the groups at day 21 after re-infection. The titer was 1/250 in both groups and the optical densities at that dilution are shown in Fig 6D.

### Normal virus propagation in epithelial cells

Because there was decreased survival and pulmonary inflammation in PAFR-deficient mice and this was associated with decreased viral load after infection with the lower inoculum at day 8 after infection, we assessed whether virus propagation was altered by the absence of PAFR. To this end, we infected A549 cell line, a human alveolar basal epithelial cell, with Influenza A virus expressing red fluorescent protein (RFP). Using this methodology, we found no difference in the ability of influenza virus to infect epithelial cells in vitro in the absence or presence of a PAFR antagonist PCA 4248 (50 µM) (data not shown).

We also measured the height of bronchiolar epithelium after infection with 10^6^ PFU to determine whether infection of epithelial cells, the primary target of influenza infection, was similar in the absence or presence of PAFR. Lethal infection promoted pronounced reduction in epithelial height of bronchiole and absence of PAFR did not alter the susceptibility of epithelial injury (Fig S3). Therefore, virus entry and replication and subsequent cell injury in its primary target, epithelial cells, is not affected when PAFR is absent.

### Activation of TLR7/8 but not TLR3 and NLRP3 was dependent of PAFR

TLR3, TLR7/8 and NALP3 are thought to be major intracellular recognition molecules used by the host to detect Influenza A infection [14]. To investigate whether these activation of these pathways could lead to inflammation in a PAFR-dependent manner, we studied the effects of synthetic agonists of TLR7/8 (R848) and TLR3 and NLRP3 [poly(I∶C)] in WT and PAFR KO mice.

**Figure 7:**
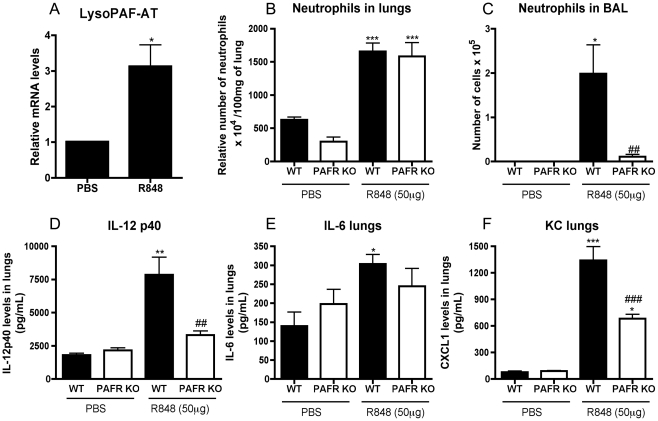
Inflammatory changes induced by R848 are PAFR dependent. WT and PAFR KO mice were instilled intratracheally with 50 µg of R848 and killed after 4 hours. Relative mRNA levels of LPAFAT/LPAFAT2 in lungs of PBS or R848 instilled WT mice assessed by Real Time PCR (A). Number of lung neutrophils, as assessed by MPO assay (B), neutrophil influx to the airways (C) after R848 instillation in WT and PAFR KO mice. Lung levels of IL-12p40 (D), IL-6 (E) and CXCL1 (F) of WT and PAFR KO mice instilled with PBS or R848 were assessed by ELISA. Data are presented as Mean ± SEM of 5–9 animals. *, ** and *** for p<0.05, p<0.01 and p<0.001, respectively, when compared to Mock groups; ## for p<0.01, and ### for p<0.001, compared to WT instilled with R848 (one-way ANOVA, Newman-Keuls).

Intratracheal instillation of R848, a TLR7/8 agonist, induced LPAFAT/LPAFAT2 mRNA in lungs of WT mice (Fig 7A). This was accompanied by infiltration of neutrophils in lungs (Fig 7B) and airways (Fig 7C), and increased levels of IL-12p40 (Fig 7D), IL-6 (Fig 7E) and CXCL1 (Fig 7F) in lungs of WT mice. In PAFR KO mice, R848 induced similar amount of neutrophil influx in lungs (Fig 7B), but greatly reduced neutrophil recruitment to the airways (Fig 7C). Levels of IL-12p40 (Fig 7D) and CXCL1 (Fig 7F) were reduced in lungs of PAFR-KO mice instilled with R848 when compared to WT.

Poly(I∶C) stimulation did not induce expression of LPAFAT/LPAFAT2 mRNA in WT mice (Fig S4A). Instillation of poly(I∶C) induced neutrophil influx in the airways (Fig S4C), but not lungs (Fig S4B) and CXCL1 production in lungs (Fig S4D) of WT mice, a response that was similar or slightly greater in PAFR KO mice.

### Treatment with a PAFR antagonist protected against Influenza A/WSN/33 H1N1 infection

**Figure 8:**
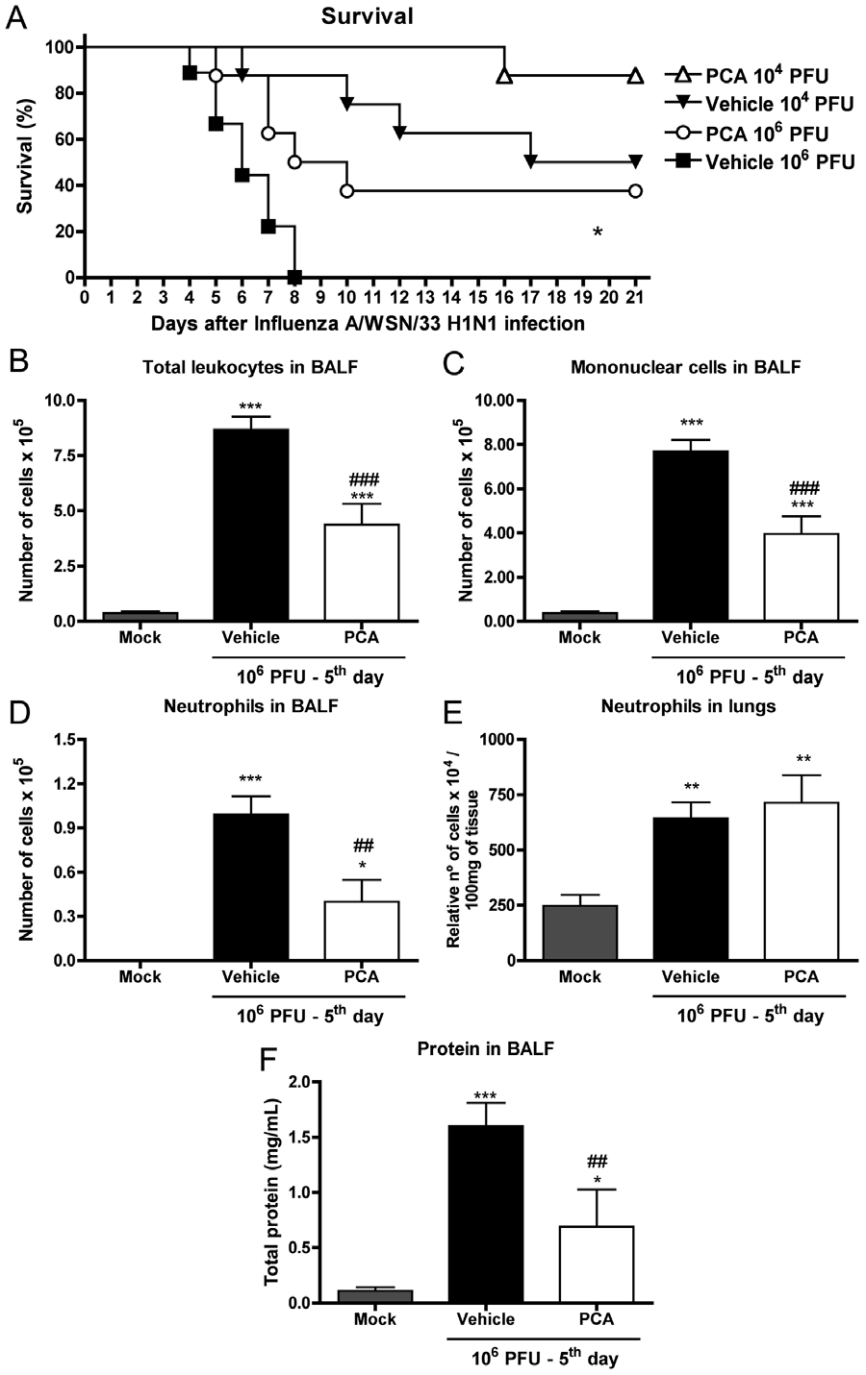
Treatment with a PAFR antagonist confers protection to mice infected with Influenza A/WSN/33 H1N1. In (A), WT mice were infected intranasally with 10^6^ PFU and 10^4^ PFU and treated twice a day, from day three to ten post infection, with vehicle or the PAFR antagonist, PCA 4248 (5 mg/kg/dose). Survival was monitored daily and was improved in PCA-treated mice infected with 10^6^ PFU, when compared to vehicle group (A); (* for p = 0.0161; log rank test, n = 8–9). To assess lung responses, mice were treated twice a day, from day three to 5 post infection, with vehicle or the PAFR antagonist, PCA 4248 (5 mg/kg/dose). Total leukocyte (B), mononuclear cell (C), and neutrophils (D) recruitment in the airways, as assessed by counts of BALF recovered cells, are shown in Mock infected and 10^6^ PFU infected, vehicle or PCA treated groups (n = 4–6, each group). Neutrophil recruitment in the lung parenchyma, as assessed by MPO (E) and total protein quantification (F) in BALF (n = 6–8, each group) are also shown. Data are presented as Mean ± SEM. *, ** and *** for p<0.05, p<0.01 and p<0.001, respectively, when compared to Mock group; ## for p<0.01 and ### for p<0.001 when compared to vehicle group (one-way ANOVA, Newman-Keuls).

To test the potential use of PAFR as a pharmacological target against Influenza A-associated disease, we used the selective PAFR antagonist PCA 4248 in a therapeutic protocol. The treatment was started 3 days after infection of mice with 10^4^ or 10^6^ PFU and was continued until day 10. Day 3 was chosen because this is the time at which weight loss and neutrophil influx peaked in the higher inoculum (Fig 1B, G). Treatment with PCA 4248 significantly enhanced survival after infection with 10^6^ PFU of Influenza A/WSN/33 H1N1 virus (Fig 8A). Akin to the experiments in PAFR KO mice, higher survival was accompanied by reduction in total leukocyte (Fig 8B), mononuclear cells (Fig 8C) and neutrophil (Fig 8D) accumulation in the airways. Neutrophilic accumulation in lung parenchyma after Influenza infection was not affected by PCA 4248 treatment (Fig 8E) as observed in PAFR KO (Fig. 2E). Plasma leakage in the airways, as assessed by measuring total protein in BAL was significantly lower in PCA 4248-treated than vehicle-treated animals (Fig 8F).

PCA treatment failed to alter significantly (from 55% to 90% survival, p = 0.10) the lethality rate caused by 10^4^ PFU infection (Fig 8A), but there was a significant decrease in weight loss from the ninth to the eleventh day of infection, when compared to the vehicle-treated group (Table 1).

**Table 1:** Body weight changes after Influenza A/WSN/33 H1N1 infection in mice treated with vehicle or PCA4246.

Day after 10^4^ PFU infection	Vehicle (% of starting weight)	PCA 4248 (% of starting weight)
**3** A	94.6±2.5 – n = 8	94.6±0.9 (p = 0.9921) – n = 8
**9**	75.8±1.9	84.2±2.6 (* p = 0.0263)
**10** B	74.8±2.6	87.4±3.2 (* p = 0.0104)
**11**	75.5±3.6	87.3±3.8 (* p = 0.0495)
**21** C	88.9±5.6 – n = 4	96.1±1.6 (p = 0.1511) – n = 7

A – First day of PCA 4248 treatment;

B – Last day of PCA 4248 treatment;

C – Last day of experiment;

*for p<0.05. Starting and finishing experimental numbers are indicated.

### PAFR deficient mice were protected from infection caused by another subtype - Influenza A H3N1

**Figure 9:**
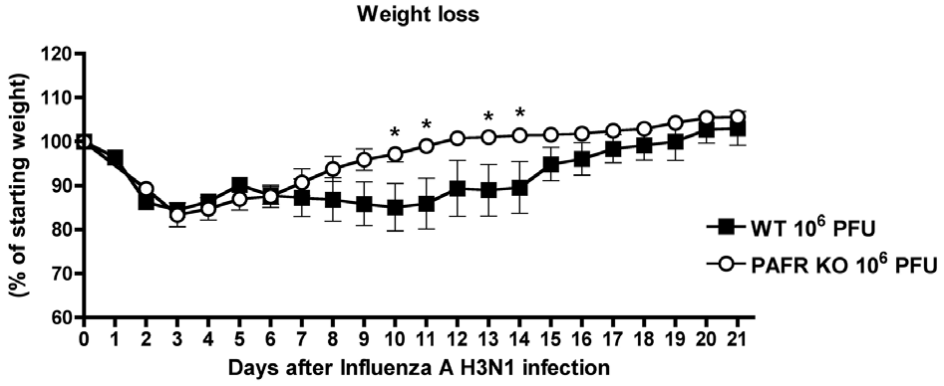
Weight loss after Influenza A (equine/Cordoba/18/1985 - Yamagata/32/1989) H3N1 infection of WT and PAFR-deficient mice. WT (n = 5) and PAFR KO (n = 6) mice were infected with intranasally 10^6^ PFU Influenza A H3N1 virus and weight monitored daily. PAFR-deficient mice lost significantly less weight at days 10, 11, 13 and 14 days after when compared to WT mice. Data are presented as Mean ± SEM. * for p<0.05. Unpaired t test.

To assess whether the protection observed in PAFR deficient mice and WT mice treated with a PAFR antagonist infected with Influenza A H1N1 was virus-specific, we used another virus strain, a reassortant Influenza A H3N1 subtype to infect WT and PAFR KO mice. Since mouse infection with different Influenza virus subtypes requires adaptation [33], H3N1 virus was subjected to three lung passages in C57BL/6J in order to cause disease in these animals. After three lung passages the virus was able to multiply and increased viral titer (data not shown), a sign of adaptation. As a result, the inoculum of 10^6^ PFU of H3N1 virus was enough to cause 15% of weight loss on average after three days of infection. PAFR KO mice were protected from the disease caused by Influenza A H3N1, since they start to regain weight, from day ten of infection, faster than WT animals (Fig 9).

## Discussion

Severe inflammation caused by highly pathogenic Influenza A strains was described to be an important cause of death during 1918 pandemics and highly pathogenic avian Influenza H5N1 infections [34]. Using a mouse-adapted Influenza A/WSN/33 H1N1 strain we show a correlation between viral load, pathogenesis and lethality – i.e. the higher the viral load, there is greater lung injury and death. More importantly, the present work demonstrates that the course of Influenza A virus infection is less severe in the absence of PAFR. Mechanistically, it appears that activation of TLR7/8 by Influenza A explains the induction of LPAFAT/LPAFAT2 mRNA and consequent activation of PAFR. Further, absence or blockade of PAFR during infection is associated with decreased neutrophil and macrophage influx into airspaces, decreased production of certain pro-inflammatory mediators and decreased lung edema, parameters which are commonly increased after pulmonary administration of PAF to rodents or humans [35], [36]. Lack of PAFR did not increase viral load or prevent specific anti-Influenza antibody production. Finally, we demonstrate that administration of PAFR antagonist 3 days after infection also causes similar protection as observed in PAFR-deficient mice.

Neutrophils and neutrophil-active chemokines peaked very early in the course of Influenza A/WSN/33 H1N1 infection and decreased thereafter. On the contrary, lung edema and injury was progressive. Thus, it seems that inflammation is self-resolving but causes lung damage which is more pronounced in the last days of infection, leading to progressive weight loss and death. As inflammation is important to trigger lung injury in Influenza A infected mice and PAF attracts neutrophils into the lung [36], we evaluated whether the receptor for PAF was involved in H1N1-associated lung inflammation and injury, and death. A previous study demonstrated up-regulation of PAFR mRNA in lungs following Influenza A/PR8/34 H1N1 infection [29]. This is consistent with the expression of PAFR on neutrophils and other leukocytes [20] and the influx of these cell types during infection. In our experiments, we found increased expression of the enzyme involved in inflammatory synthesis of PAF – LPAFAT/LPAFAT2 – in the first day of lethal infection. Therefore, the release of PAF is an early event that could be involved in the pathogenesis of Influenza A infection. In fact, PAFR deficient mice or mice treated with PAFR antagonist PCA 4248 were protected from death or weight loss caused by Influenza A virus infection. PAFR signaling was also important for pulmonary inflammation and injury.

Neutrophils are required for the clearance of Influenza virus during the early stages of infection [37]. The antiviral actions of neutrophils are clearly demonstrated through the use of RB6-8C5 antibodies to deplete these cells. In the absence of neutrophils, mice are more susceptible to virus growth and associated lethality [15], [38]. Therefore, direct ablation of neutrophils cannot be used therapeutically. On the other hand, Influenza A virus is known to be a potent activator of neutrophil respiratory burst, apoptosis and subsequent deactivation in front of a second stimulus [16]. The neutrophil activation that occurs during infection, despite its role in controlling viral replication, is thought to be very harmful to the host [37]. PAFR deficiency or antagonism increased survival after Influenza A virus infection and this was associated with lower neutrophil recruitment to the airways. There was, however, no inhibition of neutrophil into pulmonary parenchyma. Therefore, reduction instead of ablation of neutrophil recruitment into the airways appears to be an approach which is sufficient to maintain control of viral burden, but, at the same time, avoid excessive neutrophil activation and lung damage.

Bacterial pneumonia, especially by *Streptococcus pneumonia*, is an important complication following Influenza A virus infection [39]. Pro-apoptotic effects of Influenza A virus on neutrophils are one of the explanations for the increased susceptibility to bacterial pneumonia after Influenza infection [40]. In our model, absence of PAFR did not influence neutrophil apoptosis. The role of PAFR in altering bacterial pneumonia after Influenza infection is controversial. While the study of van der Sluijs and co-workers described protection in PAFR KO mice after *Streptococcus pneumoniae* following flu infection [29], McCullers and colleagues showed no correlation between the increased pathology after secondary infection and the antagonism [41] or absence of PAFR [42]. Both groups focused on lethality and harm caused by the bacteria, not the virus, and the overall message was that absence or blockade of PAFR was either without effect or protective, but never harmful. Here, we show that targeting PAFR during Influenza A virus infection is protective against viral-induced pneumonia, regardless of the risk of bacterial pneumonia after viral infection.

In addition to neutrophils, endothelial cells express PAFR and are affected by PAFR signaling [22]. Protein leakage to the airways reflects the increased vascular permeability following inflammatory events associated with Influenza A virus infection [30]. In our system, lower protein amounts or Evans' blue extravasation were found in BALF of PCA treated or PAFR KO animals. As mentioned above, neutrophil accumulation in the parenchyma was not affected by PAFR absence or antagonism. Thus, neutrophil transmigration into the alveolar space seems to play an important role in the pathogenesis of Influenza infection. During the transmigration, neutrophils may release proteinases or oxygen reactive species that lead to increase in vascular permeability that is accompanied by significant protein leakage [43]. Hence, lower protein amounts found in lethally infected mice treated with PCA or PAFR KO mice infected with lower infection inoculum compared with their controls corroborates with this hypothesis.

Pulmonary inflammation and injury are common histopathological findings in humans with mild or severe Influenza A infection. Viruses of low pathogenicity affect basically the proximal airways (bronchi and bronchioles), whereas highly pathogenic viruses or infection of immunocompromised people infected is associated with inflammation and injury in the distal airways (alveoli and parenchyma). When distal airways are affected, lung injury is more severe and lead to loss of respiratory capacity and gas exchange [44]. In our system, the extent of distal airways involvement was decreased in PAFR KO mice, suggesting these results may bear relevance to humans with severe Influenza A infection. Evaluation of gas exchange in our mice was not possible as animals needed to be anesthetized for the procedure (data not shown).

NK cells are recruited to the lungs very early in the course of Influenza infection, are involved in initial viral clearance and provide initial signals to the development of protective adaptive responses [45]. NK cells recognize Influenza A virus through its NKp46 receptor and, stimulated by IL-12 released from dendritic cells, mediate cytotoxicity to infected cells and release of IFN-γ that, in turn, mediates adaptive responses [46]. PAF seems to be important for cytotoxic functions of NK cells [47], [48] and PAFR antagonists reduced cytotoxic activity of NK cells [47]. Jin and colleagues showed that inflammation-released PAF recruits NK cells activated by IL-2, IL-12, IL-15 and IFN-α [48]. Despite these studies showing a potential effect of PAF on NK cell function and recruitment, we actually observed that NK cell recruitment to the lungs following Influenza A virus infection was increased in the absence of PAFR. Our studies do not provide a mechanism to this particular finding but do suggest that enhanced NK cell recruitment could be functionally relevant as seen by lower viral loads in the lung in some of the experiments.

In the absence of PAFR, there were reduced pulmonary levels of IFN-γ, IL-12p40 and CCL5. The reduction in these cytokines which are preferentially produced by or activate Th1 lymphocytes is related to the reduced number of CD4+ T cells in the lungs of PAFR-deficient mice infected with influenza A. These results are consistent with previous experiments in mice infected with a different microorganism, *Leishmania amazonensis*, where we also demonstrated that absence of PAFR decreased CCL5 expression and Th1-associated function [49]. However, in the case of the protozoan infection, the absence of PAFR was deleterious, because of an inability of the host to control the infection [49]. These results suggest that PAF may be important regulator of CCL5 production and consequent infiltration of effectors T cells with Th1 phenotype *in vivo*. Despite decreased IFN-γ expression and decreased accumulation of CD4+ T cells in the lungs of infected PAFR-deficient mice, viral load in the lung was decreased or at worst similar to that found in WT mice. Reduction of viral load was not associated with decreased propagation in epithelial cells *in vitro*. Furthermore, the capacity of Influenza virus to cause epithelial cell injury *in vivo* was unchanged by absence of PAFR. It is unclear why there was a small decrease in viral load in the lungs on day 8 after infection with the low inoculum. It is possible that this may reflect the better clinical status of animals and the decreased pulmonary inflammation in the absence of PAFR. Absence of PAFR was not associated with a decreased specific antibody release, as shown by re-infection studies and measurement of specific IgG titers. Therefore, changes in CD4+ T cell recruitment and related cytokines were not sufficient to modify negatively the course of infection in this model of experimental Influenza A virus infection.

Macrophages are also recruited to the lungs and airways after Influenza infection where they are thought to play an important role in the production of pro-inflammatory cytokines and in the phagocytosis of Influenza virus-induced apoptotic cells [50]. In vivo abrogation of phagocytosis of apoptotic infected cells by macrophages increases lethality rates in Influenza A infected mice [51]. We found a partial reduction of macrophage recruitment to the airways in PAFR deficient mice infected with Influenza A. There was also, a proportional reduction in number of apoptotic macrophages in PAFR deficient mice, showing that the proportion of live active macrophages was similar in both groups. These macrophages appear to be sufficient for clearance of apoptotic infected cells and helping in the resolution of the inflammatory phase of the infection [52], as seen by the reduced lung damage and survival in infected PAFR-deficient mice.

The suggestion that macrophage function in infected PAFR-deficient mice was sufficient to keep immune responsiveness is strengthened by findings that IL-6 expression was maintained and IL-1β expression was actually increased in these mice. IL-1β is produced mainly by macrophages after infection with Influenza A [13]. The role of IL-1β for viral clearance and pathology during Influenza infection is very complex [53]. Indeed, a previous study has shown that IL-1R1 deficient mice had greater lethality rates, but decreased lung damage caused by Influenza A virus infection [53]. Maines and colleagues showed a clear inverse correlation between viral virulence and IL-1β release in lungs [54]. While highly virulent H5N1 virus induces a small increase in IL-1β secretion, low virulent H5N1 stimulates high levels of this cytokine [54]. Therefore, the cytokine IL-1β does appear to contribute to viral clearance, even at the cost of increasing pulmonary recruitment of leukocytes in some models. In our system, enhanced release of IL-1β in PAFR-deficient mice was associated with maintained or decreased viral load, but no enhancement of leukocyte infiltration or pathology. The inability of IL-1β to enhance inflammation in the system may be explained by the role of PAFR in mediating IL-1β-associated inflammation [55], [56].

The recognition of Influenza virus RNA is the main trigger of antiviral and inflammatory responses [14]. Using the synthetic TLR agonist R848, we found that activation of this ssRNA sensor induces the expression of LPAFAT/LPAFAT2 and inflammation that is directly dependent of PAFR. It has been previously shown that LPAFAT/LPAFAT2 expression and enzyme activity may be induced by the TLR9 ligand ODN1826 and the TLR4 ligand LPS [19]. Poly(I∶C), a classical TLR3 ligand and recently described as a NLRP3 activator [10], did not induce LPAFAT/LPAFAT2 mRNA expression and cause pulmonary inflammation which was PAFR-independent. Therefore, of the known major recognition receptors for Influenza, it appears that TLR7/8 is the one capable of inducing LPAFAT/LPAFAT2 expression and triggers pulmonary inflammation in PAFR-dependent manner. Results with R848 were qualitatively similar (LPAFAT/LPAFAT2 mRNA expression, PAFR-dependency of inflammation) to that observed after Influenza infection, suggesting that activation of TLR7/8 is a main mechanism by which Influenza infection leads PAF release and PAFR activation. One alternative possibility to explain the activation of PAFR in our system derives from recent findings that oxidized phospholipids (Ox-PL) were produced during acute lung injury induced by inactivated H5N1 [27]. Ox-PL are PAF-like molecules known to induce PMN migration and protein leakage in pleural cavity, effects which are PAFR-dependent [57]. Ox-PLs levels were also correlated with the reduced severity of flu in IL-17RA KO mice infected with Influenza A/PR/8/34 (H1N1) [28]. Thus, production of Ox-PL may contribute to the activation of PAFR during influenza infection.

The present model of Influenza A/WSN/33 H1N1 infection mimics infections with highly pathogenic strains during pandemics or in immunocompromised people. We used a low inoculum of H1N1 and a low pathogenic Influenza A strain to model the common features of the seasonal flu in humans. Using a low pathogenic Influenza A H3N1 reassortant virus, we showed that PAFR deficient mice had decreased weight loss in comparison to WT mice. Therefore, PAF may be important to the pathogenesis of Influenza A virus infection, regardless of subtype or strain. This feature could be explained because PAFR targeting modulates the inflammatory response to the virus, without affecting, or also improving viral clearance by the host. Recently, our group published similar results in a murine model of Dengue virus infection. In that work, the absence or pharmacological blockade of PAFR resulted in protection against the main symptoms of dengue virus infection and lethality, regardless of viral titer change [58].

In conclusion, our studies clearly show that PAFR-mediated inflammatory events that follow Influenza A virus infection are important for disease pathogenesis and lethality. Mechanistically, protection in PAFR deficient mice was associated with decreased infiltration of neutrophils and macrophages into the airways and decreased lung damage. Importantly, PAFR deficiency tended to enhance the ability of the murine host to deal with the virus and antibody and adaptive response were maintained. Importantly, treatment with PAFR antagonists starting 3 days after infection also protected against Influenza A morbidity and lethality. These studies show that PAFR is a disease-associated gene after Influenza A virus infection and suggest that PAFR antagonism could be a useful therapeutic target to interfere with inflammatory damage that follows infection. It remains to be determined whether this will be a useful therapeutic strategy in humans and whether association with antiviral will enhance benefits provided by each individual strategy, as suggested elsewhere [59].

## Methods

### Ethics statement

All the experiments were conducted under prior CETEA/UFMG animal ethics committee approval (203/08), according to Brazilian guidelines on animal work.

### Virus strains and culture

The strains used in the study were Influenza A WSN/33 H1N1 and Influenza A (H3 of equine/Cordoba/18/1985 and N1 of Yamagata/32/1989) H3N1. Briefly, Influenza A WSN/33 H1N1 was produced in chicken eggs and passed once more in eggs and then cultured in MDCK (Madin-Darby Canine Kidney) cells. The stocks in a final concentration between 4×10^7^ and 2×10^8^ PFU/mL were diluted in sterile phosphate buffered saline prior to infections.

Influenza A H3N1 strain was adapted to mice through three lung passages. Briefly, 10^4^ PFU of Influenza A H3N1virus was inoculated via intranasal in five animals and after 5 days lungs were collected an tittered. The sample that had the higher titer (10^5^ PFU) was passed through a 0.45 µm filter and used to infect another 5 animals (100 PFU/animal). The process of selection was repeated once and the final titer achieved was 5×10^7^ PFU. The stock was diluted in sterile phosphate buffered saline prior to infections.

### Animal infections

Male 8–10 weeks C57BL/6J and PAFR deficient mice (PAFR KO) on a C57BL/6J background, maintained in pathogen free conditions at Laboratorio de Imunofarmacologia (UFMG/Brazil) facilities, were used in the infection experiments. Mice anesthetized with ketamine/xylazine received 25µL of Influenza A/WSN/33 H1N1 virus, Influenza A H3N1virus, or sterile phosphate buffered saline (PBS, Mock group), intranasally. The H3N1 virus was previously adapted to mice, through three lung passages as described above.

### Drug treatment

The PAFR antagonist PCA 4248 (Tocris Bioscience), 5mg/kg, diluted in 5% of ethanol in PBS was given twice a day, via subcutaneous injections. The control group (vehicle) received the same volume of the solution used to dilute PCA 4248.

### In vitro infections

MDCK and the human lung epithelial cell line A549 (ATCC CCL-185) were cultured in Dulbecco's Modified Eagle Medium (DMEM) supplemented with 6% of fetal bovine serum were cultured at 37°C with 5% of CO_2_. Cells were seeded at a density of 4×10^4^ cells/well in a 24-well plate and after 24 hours of growth were incubated with 50 µM of PCA 4248 in DMEM or vehicle for 30 minutes. Cells were infected with a multiplicity of infection of 2 with a red fluorescent protein (RFP) labeled Influenza A virus and incubated for 16 hours. Virus propagation was observed through a fluorescence microscopy.

### Experimental design

#### Influenza A infection

Wild Type (WT) mice were infected with distinct Influenza A/WSN/33 H1N1 inocula. PAFR KO and WT mice, PCA 4248 or vehicle treated were infected with lethal (10^6^ PFU) and lower infection (10^4^ PFU) inocula. Mice were monitored by weight loss and lethality for 21 days or sacrificed after indicated days of infection to have cellular recruitment to the airways and to the lungs evaluated, as well as cytokine and chemokine production, and lung injury and viral load assessment. Animals which lost more than 30% of their initial weight were killed; however, the maximum weight loss observed was lower than 30%. LysoPAFAT/LPCAT2 gene expression was assessed in lungs of WT mice infected with the lethal inoculum. WT and PAFR KO mice were infected with the non-lethal inoculum of 10^3^ PFU of Influenza A/WSN/33 H1N1, monitored for 14 days and reinfected with the lethal inoculum of the same virus. After 21 days, reinfected mice were killed and serum samples were used to measure specific IgG titers. Finally, WT and PAFR KO mice were infected with Influenza A (equine/Cordoba/18/1985 - Yamagata/32/1989) H3N1 and monitored for 21 days.

#### TLR7/8, TLR3 and NLRP3 activation

Anesthetized WT and PAFR KO mice were instilled intratracheally with 40µL containing a sterile PBS with 50 µg of R848 or poly(I∶C) (Invivogen, San Diego, USA), or saline only (controls) and killed at 4 (R848) or 8 [poly(I∶C)] h after instillation. Lungs were washed and removed for evaluation of cell, MPO levels, and cytokine and chemokine production. The lower lobe of the left lung of WT animals was excised for the evaluation of LysoPAFAT/LPCAT2 gene expression by Real Time PCR.

### Bronchoalveolar lavage (BAL)

At indicated time points, mice were euthanized with an overdose of ketamine/xylazine solution. Subsequently, a 1.7mm catheter was inserted into the trachea and two 1mL aliquots of PBS were inserted into the lungs and collected three times to acquire leukocytes recruited to the airway space. After centrifugation, the pellet was used to total and differential leukocytes counts of stained slides.

### Assessment of pulmonary vascular leakage by Evans blue

At day 5 after infection with 10^6^ PFU of Influenza A WSN/33 or PBS instillation, WT and PAFR were injected with Evans blue dye (50 mg/kg, i.v.) 2 h before they were killed. Animals were subjected to BAL with 1mL of PBS. BALF was then centrifuged and the optical density was determined at 620 nm. The concentration of extravasated EBD (microgram of EBD per gram lung) in lung homogenates was calculated against a standard curve.

### Lung myeloperoxidase measurement

After performing BAL, lungs were perfused with 5 mL of PBS to remove circulating blood and frozen. A hundred µl of tissue was homogenized to perform ELISA and MPO assay, as previously described [60].

### Measurement of cytokines and chemokines

Lung tissues were homogenized in a PBS-buffer containing antiproteases, as previously described [60], to assess the concentrations of the cytokines IL-1β, IL-6, IL-12 p40, IFN-γ and TNF-α and the chemokines CXCL1, CXCL2, CCL2, CCL5 and serum IL-6 levels by ELISA DuoSet kits (R&D Systems), in accordance to the manufacturer's instructions.

### Real time PCR

Total RNA from diaphragmatic lung lobe tissue conserved at −70°C in RNA later (Applied Biosystems, California, USA) was extracted using Trizol (Invitrogen), as described by the manufacturer. The total RNA obtained was suspended in RNAse-free water and stocked at −70°C. Real-time PCR was performed on an ABI PRISM Step-One sequence-detection system (Applied Biosystems) by using SYBR Green PCR Master Mix (Applied Biosystems) after a reverse transcription reaction of 2 µg of RNA by using M-MLV reverse transcriptase (Promega). The relative expression level of LysoPAFAT/LPCAT2 gene was determined by the 2 (−delta delta Ct) method, normalized by ribosomal subunit 18S and expressed as fold change compared with constitutive gene expression. The following primer pairs were used: LysoPAFAT/LPCAT2 forward 5′ GTCCAGCAGACTACGATCAGTG 3′; LysoPAFAT/LPCAT2 reverse 5′ CTTATTGGATGGGTCAGCTTTTC 3′ as described by [19]; and 18S forward 5′ CTCAACACGGGAAACCTCAC 3′; 18S reverse 5′ CGTTCCACCAACTAAGAACG 3′.

### Assessment of lung injury and histological analysis

We analyzed lung injury following Influenza A virus infection assessing protein leakage to the airways, histological changes in lung architecture. A protein quantification assay (Bio-Rad Protein Assay) was performed in bronchoalveolar lavage fluid (BALF) supernatant according to manufacturer's instructions. Formalin-fixed lung left lobes were dehydrated gradually in ethanol, embedded in paraffin, cut into 4-µm sections, stained with H&E and examined under light microscopy and scored by a pathologist blinded to the experiment. The score of 18 points was based on Horvat and colleagues paper [61], which evaluates airway, vascular and parenchymal inflammation, added to a 5 points score evaluating general neutrophilic infiltration (0, absent; 1, minimal; 2, slight; 3, moderate; 4, marked; and 5, severe).

Photographs of areas containing bronchioles in H&E stained slides were taken under 200 fold of magnification and were analyzed with an AxioVision software. Epithelial height of bronchioles in areas of inflammatory infiltrates had their length measured. The number of bronchioles analyzed in each slide varied according to their length, to totalize 1500 µm per slide. The mean epithelial height for each animal was used to construct the graph.

### Flow-cytometric analysis of lung and airway leukocyte populations

Leukocytes recovered from BALF and from lungs processed with collagenase IV (Sigma) [62] were stained with fluorescent-labeled monoclonal antibodies CD3, CD4, CD8, NK1.1 and GR1 (BD Pharmigen TM); CXCR2 (R&D Systems); F4/80 and CCR5 (Biolegend). Stained cells were acquired in FACScan cytometer and analyzed in FlowJo (Tree Star) software (Text S1).

### Apoptosis assay

We performed Annexin V binding assay on BALF leukocytes using Annexin V/Propidium Iodide (PI) kit (Caltag Laboratories) according to manufacturer's instructions. Flow cytometry was carried out in FACScan and analyzed in FlowJo software.

### Plaque assay

In order to determine viral load in lungs, we collected the organs in sterile conditions and performed a plaque assay using MDCK cells. Lungs were weighted and homogenized in PBS, plated in MDCK monolayer and after incubation and staining it was possible to count the plaques. The viral titer was expresses as Plaque Forming Units (PFU) per gram of tissue.

### Antibody quantification

WSN/33 H1N1 influenza virus stocks were used as antigens in an indirect ELISA to detect specific antibodies in serum samples of reinfected animals (Text S1).

### Statistical analysis

All data are presented as the mean ± SEM. All data were tested for normality and found to have a normal distribution. Normal data were tested for significance using ANOVA followed by use of Newman-Keuls post-test, which corrects for multiple comparisons. Unpaired t test was used to compare two groups and Log-rank test for lethality experiments, where appropriate. Statistical significance was set as P<0.05 and all graphs and analysis were performed using Graph Pad Prism 4 software.

## Supporting Information

Figure S1 Pulmonary levels of inflammatory cytokines and chemokines following mild Influenza A/WSN/33 H1N1 infection. Mice were infected intranasally with 10^4^ PFU of Influenza virus or PBS (Mock) and killed 1, 4, 7 and 10 days after infection (n = 4–10 in each group). Pulmonary levels of CXCL1/KC (a), CXCL2/MIP-2 (b), CCL2/MCP-1 (c) and TNF-α (d) were assessed by ELISA. Data are presented as Mean ± SEM. *, ** and *** for p<0.05, p<0.01 and p<0.001, respectively, when compared to Mock or indicated groups, (one-way ANOVA, Newman-Keuls).
(0.21 MB TIF)

Figure S2 Pulmonary levels of inflammatory cytokines and chemokines following lethal Influenza A/WSN/33 H1N1 infection. Mice were infected intranasally with 10^6^ PFU of Influenza virus or PBS (Mock) and killed 1, 3 and 5 days after infection (n = 6–7 in each group). Pulmonary levels of CXCL1/KC (a), CXCL2/MIP-2 (b), CCL2/MCP-1 (c), TNF-α (d) were assessed by ELISA. Data are presented as Mean ± SEM. *, ** and *** for p<0.05, p<0.01 and p<0.001, respectively, when compared to Mock or indicated groups; # for p<0.05, when compared to Mock group (one-way ANOVA, Newman-Keuls).
(0.19 MB TIF)

Figure S3 Mean epithelial height of bronchiole following lethal Influenza A/WSN/33 H1N1 infection. WT and PAFR KO mice were infected intranasally with 10^6^ PFU of Influenza virus or PBS (Mock) and were killed after 5 days of infection. H&E stained lung slides were photographed under 200 fold magnification. A total of 1500 µm of bronchiolar length in areas of inflammatory infiltrates per slide was divided in 50 µm. In every each 50 µm epithelial height was measured. Results present the mean of the measures of 5–6 animals. Data are presented as Mean ± SEM. ** p<0.01, when compared to Mock groups (one-way ANOVA, Newman-Keuls).
(0.11 MB TIF)

Figure S4 Inflammatory changes induced by poly(I∶C) are not PAFR dependent. WT and PAFR KO mice were instilled intratracheally with 50 µg of poly(I∶C) and killed after 8 hours. Relative mRNA levels of LPAFAT/LPAFAT2 in lungs of PBS or poly(I∶C) instilled WT mice assessed by Real Time PCR (a). Number of lung neutrophils, as assessed by MPO assay (b), neutrophil influx to the airways (c) and CXCL1 levels in lungs (d) of WT and PAFR KO mice instilled with PBS or poly(I∶C). Data are presented as Mean ± SEM of 5–7 animals. *, ** and *** for p<0.05, p<0.01 and p<0.001, respectively, when compared to Mock groups; (one-way ANOVA, Newman-Keuls).
(0.27 MB TIF)

Text S1 Methods and legends for supporting figures.
(0.11 MB PDF)
