# Supplementary figures and images for: Platelet-Activating Factor Receptor Plays a Role in Lung Injury and Death Caused by Influenza A in Mice
Source: PLoS Pathog. 2010 Nov 4;6(11):e1001171. doi: 10.1371/journal.ppat.1001171 (PMC2974216; doi:10.1371/journal.ppat.1001171)

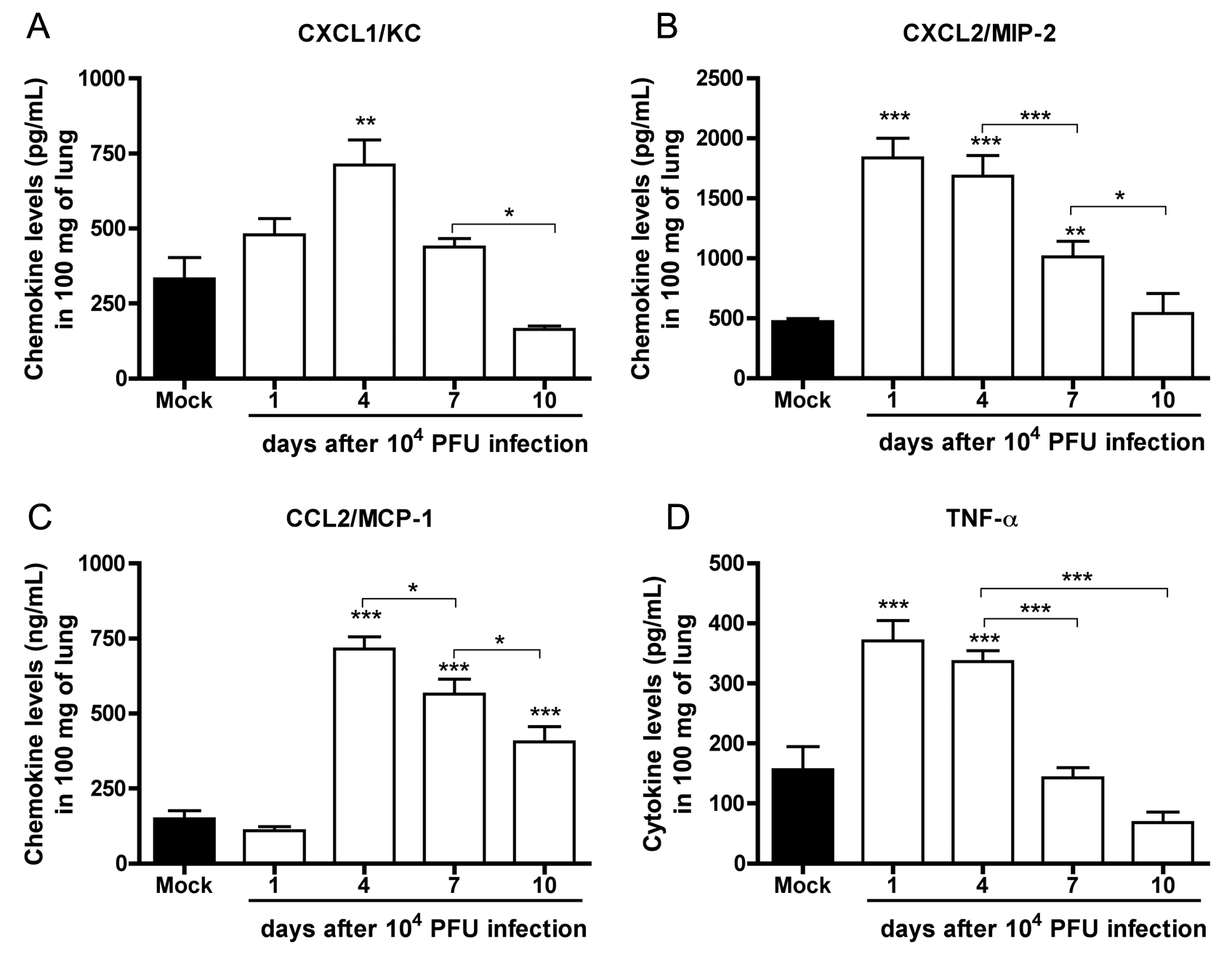

Supplement: Figure S1 — Pulmonary levels of inflammatory cytokines and chemokines following mild Influenza A/WSN/33 H1N1 infection. Mice were infected intranasally with 104 PFU of Influenza virus or PBS (Mock) and killed 1, 4, 7 and 10 days after infection (n = 4–10 in each group). Pulmonary levels of CXCL1/KC (a), CXCL2/MIP-2 (b), CCL2/MCP-1 (c) and TNF-α (d) were assessed by ELISA. Data are presented as Mean ± SEM. *, ** and *** for p<0.05, p<0.01 and p<0.001, respectively, when compared to Mock or indicated groups, (one-way ANOVA, Newman-Keuls). (0.21 MB TIF) [file ppat.1001171.s001.tif]

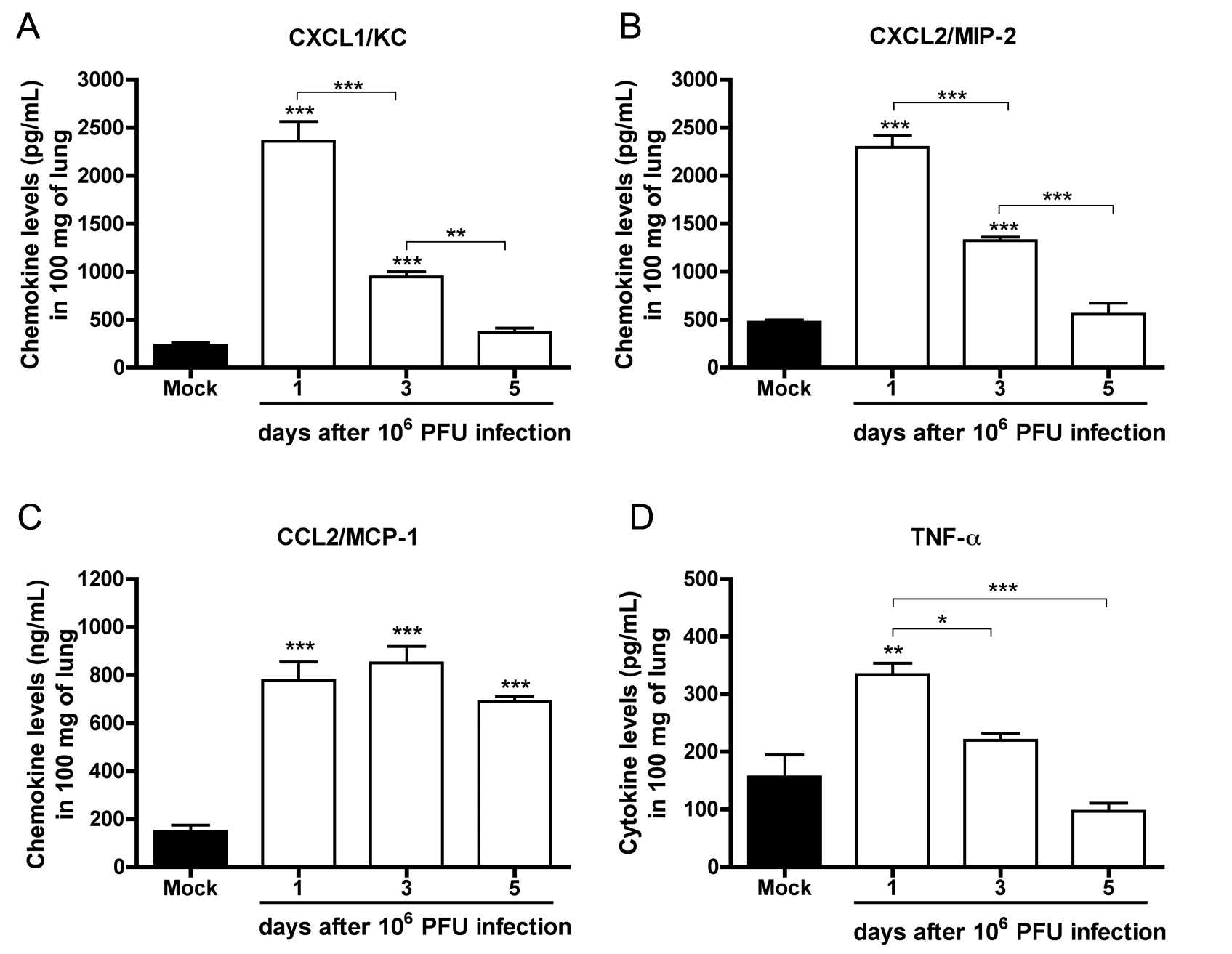

Supplement: Figure S2 — Pulmonary levels of inflammatory cytokines and chemokines following lethal Influenza A/WSN/33 H1N1 infection. Mice were infected intranasally with 106 PFU of Influenza virus or PBS (Mock) and killed 1, 3 and 5 days after infection (n = 6–7 in each group). Pulmonary levels of CXCL1/KC (a), CXCL2/MIP-2 (b), CCL2/MCP-1 (c), TNF-α (d) were assessed by ELISA. Data are presented as Mean ± SEM. *, ** and *** for p<0.05, p<0.01 and p<0.001, respectively, when compared to Mock or indicated groups; # for p<0.05, when compared to Mock group (one-way ANOVA, Newman-Keuls). (0.19 MB TIF) [file ppat.1001171.s002.tif]

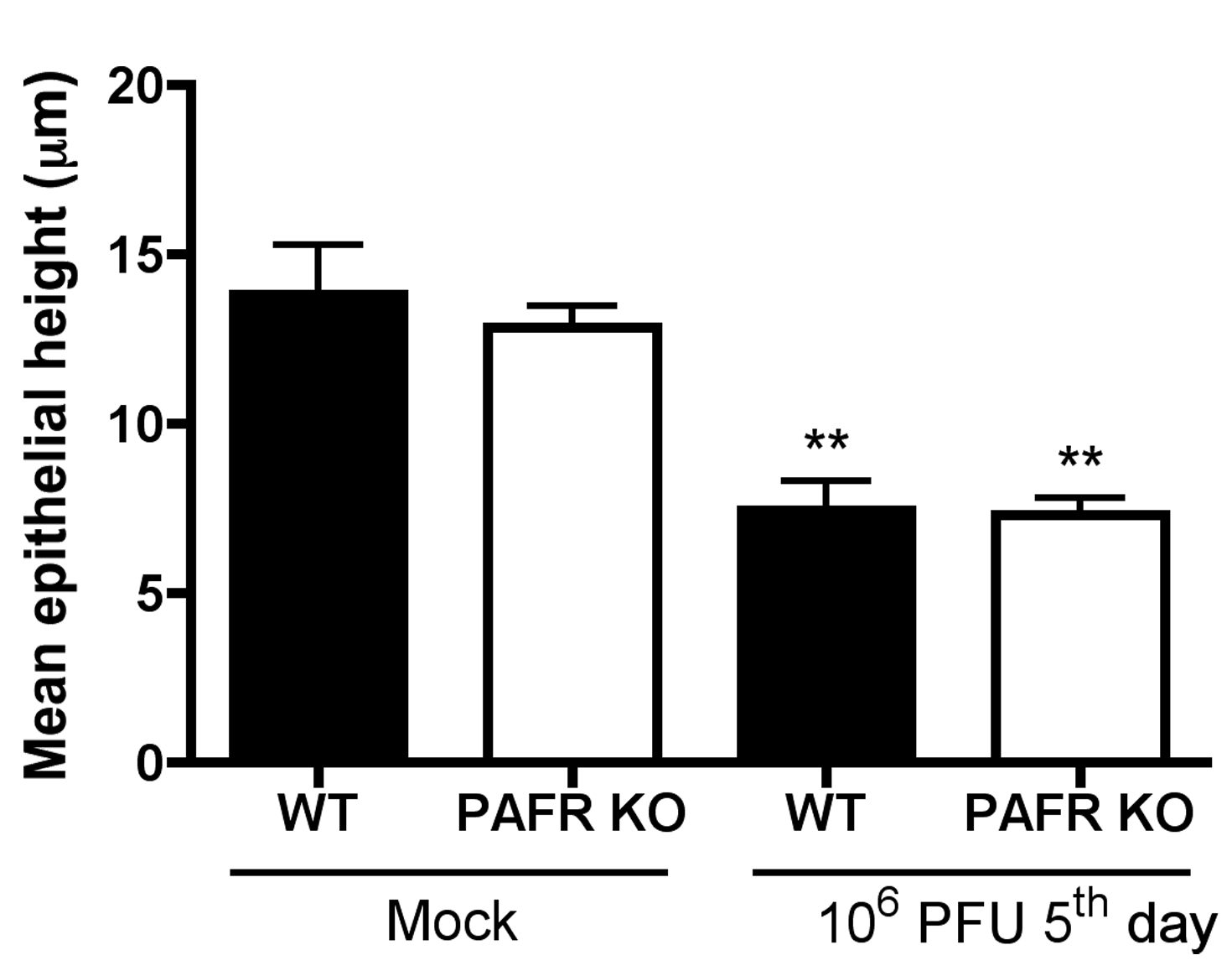

Supplement: Figure S3 — Mean epithelial height of bronchiole following lethal Influenza A/WSN/33 H1N1 infection. WT and PAFR KO mice were infected intranasally with 106 PFU of Influenza virus or PBS (Mock) and were killed after 5 days of infection. H&E stained lung slides were photographed under 200 fold magnification. A total of 1500 µm of bronchiolar length in areas of inflammatory infiltrates per slide was divided in 50 µm. In every each 50 µm epithelial height was measured. Results present the mean of the measures of 5–6 animals. Data are presented as Mean ± SEM. ** p<0.01, when compared to Mock groups (one-way ANOVA, Newman-Keuls). (0.11 MB TIF) [file ppat.1001171.s003.tif]

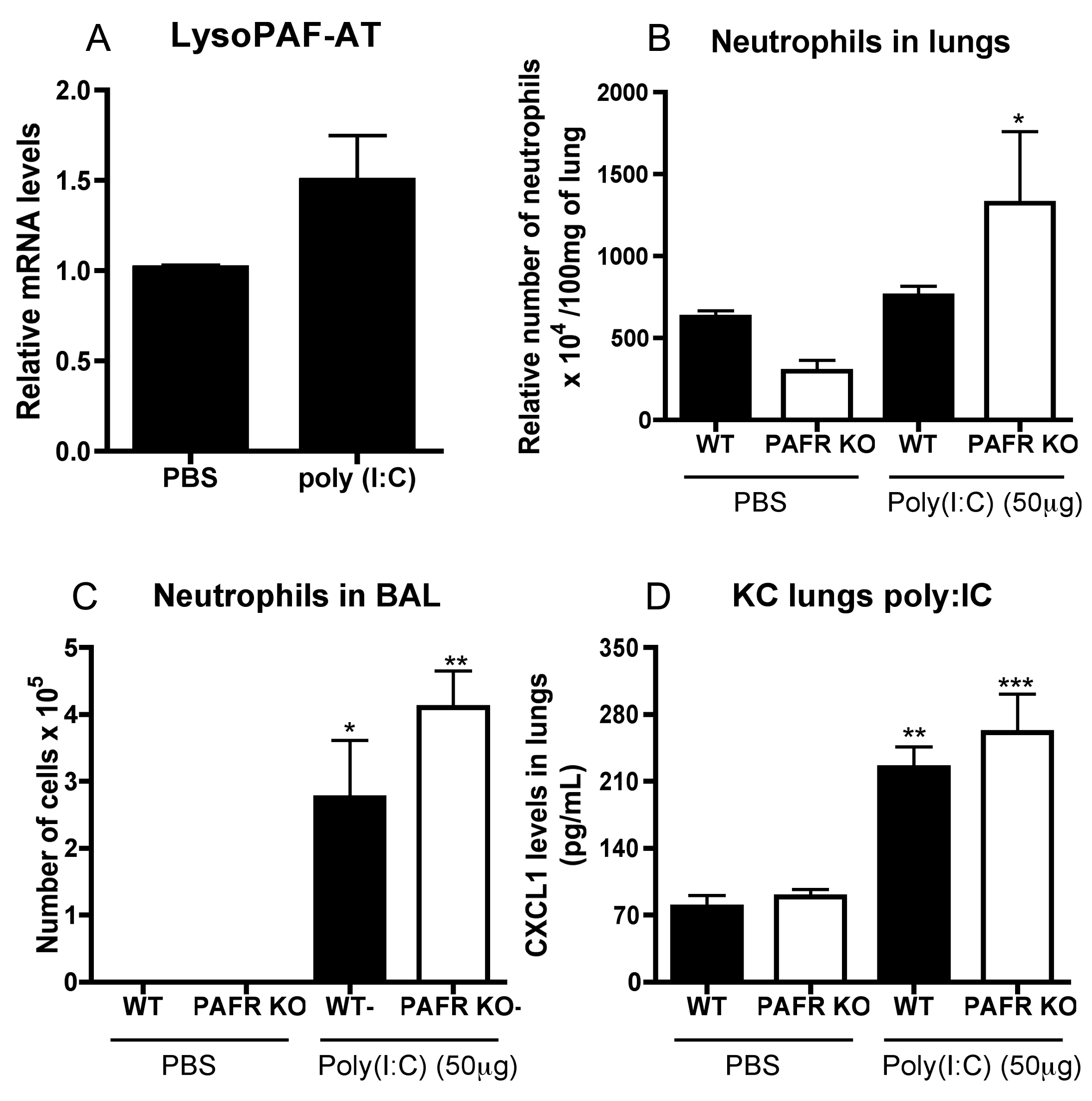

Supplement: Figure S4 — Inflammatory changes induced by poly(I∶C) are not PAFR dependent. WT and PAFR KO mice were instilled intratracheally with 50 µg of poly(I∶C) and killed after 8 hours. Relative mRNA levels of LPAFAT/LPAFAT2 in lungs of PBS or poly(I∶C) instilled WT mice assessed by Real Time PCR (a). Number of lung neutrophils, as assessed by MPO assay (b), neutrophil influx to the airways (c) and CXCL1 levels in lungs (d) of WT and PAFR KO mice instilled with PBS or poly(I∶C). Data are presented as Mean ± SEM of 5–7 animals. *, ** and *** for p<0.05, p<0.01 and p<0.001, respectively, when compared to Mock groups; (one-way ANOVA, Newman-Keuls). (0.27 MB TIF) [file ppat.1001171.s004.tif]
